# Cerebral ischemic damage in diabetes: an inflammatory perspective

**DOI:** 10.1186/s12974-016-0774-5

**Published:** 2017-01-23

**Authors:** Vibha Shukla, Akhalesh Kumar Shakya, Miguel A. Perez-Pinzon, Kunjan R. Dave

**Affiliations:** 1 Cerebral Vascular Disease Research Laboratories, University of Miami School of Medicine, Miami, FL 33136 USA; 2 Department of Neurology (D4-5), University of Miami Miller School of Medicine, 1420 NW 9th Ave, NRB/203E, Miami, FL 33136 USA; 3 Neuroscience Program, University of Miami School of Medicine, Miami, FL 33136 USA; 4 Present address: Department of Microbiology and Immunology, and Center for Molecular and Tumor Virology, Louisiana State University Health Sciences Center, Shreveport, LA 71130 USA

**Keywords:** Inflammation, Stroke, Hypoglycemia, Hyperglycemia, Cell death, Diabetic brain, Cytokines, Chemokines, Immune cells

## Abstract

Stroke is one of the leading causes of death worldwide. A strong inflammatory response characterized by activation and release of cytokines, chemokines, adhesion molecules, and proteolytic enzymes contributes to brain damage following stroke. Stroke outcomes are worse among diabetics, resulting in increased mortality and disabilities. Diabetes involves chronic inflammation manifested by reactive oxygen species generation, expression of proinflammatory cytokines, and activation/expression of other inflammatory mediators. It appears that increased proinflammatory processes due to diabetes are further accelerated after cerebral ischemia, leading to increased ischemic damage. Hypoglycemia is an intrinsic side effect owing to glucose-lowering therapy in diabetics, and is known to induce proinflammatory changes as well as exacerbate cerebral damage in experimental stroke. Here, we present a review of available literature on the contribution of neuroinflammation to increased cerebral ischemic damage in diabetics. We also describe the role of hypoglycemia in neuroinflammation and cerebral ischemic damage in diabetics. Understanding the role of neuroinflammatory mechanisms in worsening stroke outcome in diabetics may help limit ischemic brain injury and improve clinical outcomes.

## Background

### Diabetes

Diabetes is one of the most important metabolic disorders for public health owing to the increased prevalence of diabetes cases worldwide. According to the International Diabetes Federation, there are 382 million people living with diabetes worldwide [1]. The World Health Organization estimates that in 2030, diabetes will be the seventh leading cause of death [2]. Diabetes occur due to insufficient production of insulin or/and improper action of insulin (http://www.who.int/mediacentre/factsheets/fs312/en/) (http://www.who.int/mediacentre/factsheets/fs312/en/). Type 1 and type 2 are the major types of diabetes (http://www.who.int/mediacentre/factsheets/fs312/en/). Type 1 diabetes (T1D) is characterized by loss of pancreatic β cells whereas type 2 diabetes (T2D) is the consequence of decreased insulin response (resistance) which in later stages is accompanied by failure of pancreatic β cells [3, 4].

### Glucose-lowering drugs and risk of hypoglycemia

During the last decades, the intensive use of insulin or other drugs, which stimulates insulin secretion, as the main treatment to prevent hyperglycemia and its long-term complications has resulted in an increase in the incidence of hypoglycemia in diabetic patients [5]. An intensively treated individual with T1D can experience up to 10 episodes of symptomatic hypoglycemia per week and severe temporarily disabling hypoglycemia at least once a year (reviewed in [6]). In addition, an impaired counter-regulatory response results in frequent episodes of hypoglycemia in diabetic patients [7, 8]. However, hypoglycemia becomes progressively more frequent, depending upon the history of hypoglycemia and the duration of insulin treatment [9, 10]. Hypoglycemia is estimated to account for about 2–4% of deaths in T1D patients [11]. In a study among young patients with T1D, continuous glucose monitoring (CGM) has revealed frequent and prolonged asymptomatic (glucose <65 mg/dl) hypoglycemia in almost 70% of patients [12]. A similar study in relatively older T1D patients observed that these patients experience hypoglycemia (glucose ≤70 mg/dl) for an average of 60–89 min/day, or 4–6% of the time [13].

The increased prevalence of hypoglycemia has also been noticed in a more recent study on T2D using the CGM system [14]. In this study on 108 T2D patients were monitored for 5 days, CGM system revealed that 49% of patients had a mean of 1.74 episodes/patient during observation period and 75% of those patients experienced at least one asymptomatic hypoglycemic episode during observation period. High prevalence of hypoglycemia (82% had at least one hypoglycemic event) has been noticed by another study that monitored T2D patients for 72-h monitoring using the CGM system [15]. T2D patients are known to suffer from several episodes of asymptomatic hypoglycemia every week, symptomatic hypoglycemia as frequently as twice per week, and experience one episode of severe (episodes that require assistance of another individual) hypoglycemia per year [16].

Hypoglycemia is a threatening condition, as normal brain functioning is highly dependent on a continuous supply of glucose from the blood [17]. Episodes of hypoglycemia can include symptoms such as warmth, weakness and fatigue, difficulty in thinking, confusion, behavioral changes, and emotional lability. Seizures and loss of consciousness are observed during severe hypoglycemia. In more severe cases, brain damage and even death are possible [17].

The most common cause of hypoglycemia is intensive glycemic control, the involuntary intake of excessive doses of insulin or other glucose-decreasing drugs, or hypoglycemia unawareness [16]. Skipping meals, eating smaller meals, and having an irregular eating pattern are also known risk factors for hypoglycemia. Children with T1D are at higher risk of hypoglycemia due to difficulty in insulin dosing, unpredictable activity and ﻿ eating patterns, and limitations in detecting hypoglycemia in this population [18]. Variety of other factors such as aging, patients with vascular disease or renal failure, pregnant women, and young T1D patients also contributes to the high risk of hypoglycemia [5, 18]. In T2D individuals, the risk of hypoglycemia gradually increases due to progressive insulin deficiency, longer duration of diabetes, and tight glycemic control (reviewed in [19]). Hypoglycemia is known to cause neurologic deficits ranging from reversible focal deficits to irreversible coma. The associated neurologic deficits can be attributed to cerebral “excitotoxic” neuropathologies, where neurons selectively die due to an extracellular overflow of excitatory amino acids produced by the brain itself [20, 21]. Severe hypoglycemia can lead to brain damage when accompanied by the silencing of the brain activity (electroencephalographic isoelectricity or hypoglycemic coma) [22, 23]. Impairment in learning and memory has been reported in animals suffering from hypoglycemic coma, which correlates with neuronal damage in the hippocampus [24]. Cognitive dysfunction has also been reported in diabetic children and adults with poor glycemic control after experiencing acute hypoglycemia [25–28]. Although moderate hypoglycemia is not life-threatening, if recurrent, it may have serious clinical implications. The presence of oxidative stress and neuronal death during hypoglycemia has been documented previously by several investigators [29–31]. Hypoglycemia can also activate inflammation by increasing the plasma level of P-selectin, an adhesion molecule that is activated by inflammation [32].

### Diabetes and secondary complications

Long-term diabetes results in secondary complications of diabetes. Many health issues stem from this disease including heart disease, increased risk of stroke, hypoglycemia, vision loss, kidney failure, amputations, and complications within the central nervous system (CNS) (reviewed in detail in [33, 34]). Manifestations of diabetes-induced CNS complications may include structural alterations or brain atrophy, as well as changes in electrophysiological properties that ultimately result in deficits in cognitive performance [35].

### Diabetes and risk of cerebral ischemia

Diabetes increases the risk of cerebral ischemia either by ischemic stroke or cardiovascular diseases (CVD) [36, 37]. In most animal studies, acute hyperglycemia immediately before or during ischemia exacerbates the ischemic brain injury [38–41]. Meta-analysis of prospective studies showed a hazard ratio of 2.27 for ischemic stroke in diabetics compared to non-diabetics [42]. Diabetes and diabetes-associated risk factors contribute to atherosclerotic changes in the heart and the cerebropetal arteries. They are also associated with an increased risk of different subtypes of ischemic stroke (including lacunar, large artery occlusive, and thromboembolic strokes) [43–45]. Meta-analysis of prospective cohort and case-control studies of diabetes and risk of atrial fibrillation showed that diabetes is associated with an increased risk of subsequent atrial fibrillation, which is a major cause of thromboembolic stroke. The risk is increased by 40% in individuals with diabetes [46].

### Diabetes and aggravation of cerebral ischemic damage

**Fig. 1:**
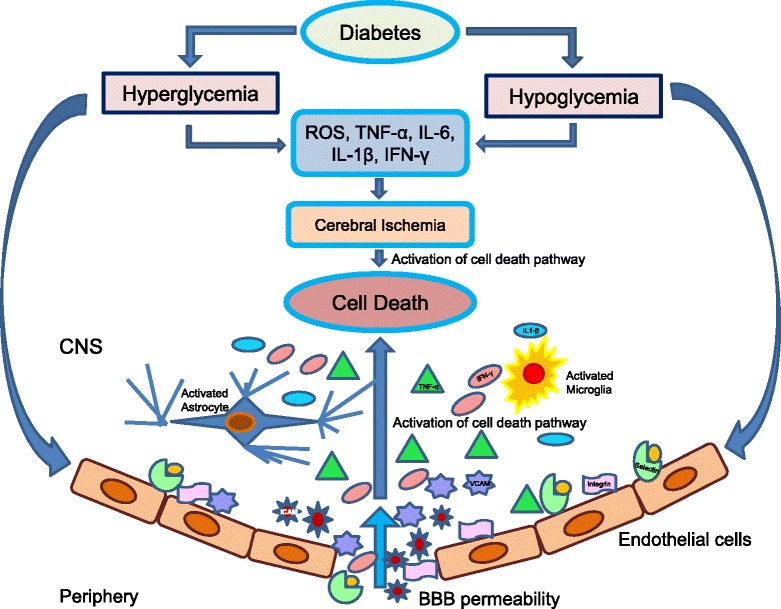
Schematic representation of neuroinflammatory mechanisms involved in aggravating brain damage following cerebral ischemia under hyperglycemic/hypoglycemic conditions

**Fig. 2:**
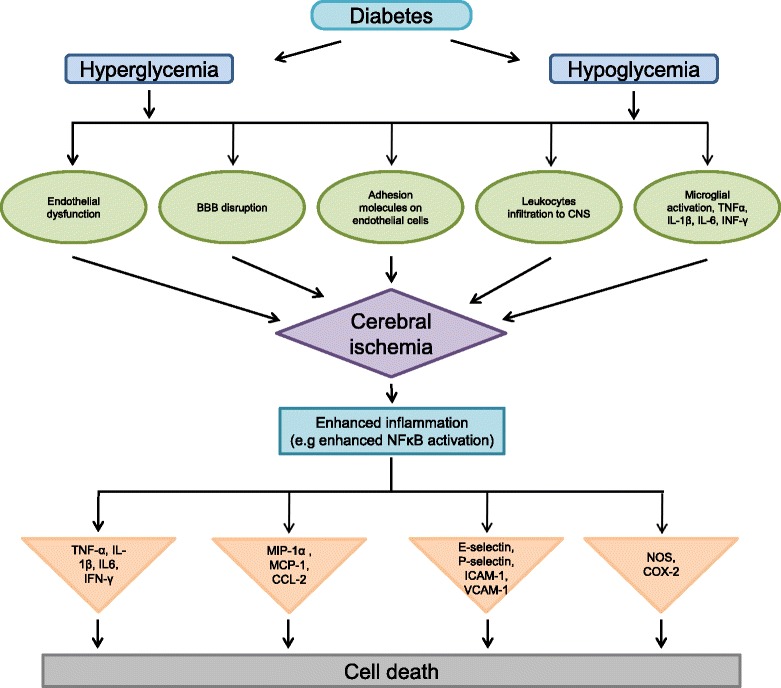
Detailed schematic representation of neuroinflammatory mechanisms involved in aggravating brain damage following cerebral ischemia in diabetes (hyperglycemic and hypoglycemic conditions)

Ischemic stroke results from the obstruction of blood flow of an artery within the brain, leading to cell death and infarction accounting for about 87% of all strokes [47]. Ischemia leads to irreversible brain damage. In addition, hypoglycemia and diabetes have been reported to aggravate damage following cerebrovascular disorder owing to the involvement of many deleterious pathways including oxidative stress, impaired leukocyte function, abnormal angiogenesis, increased blood–brain barrier (BBB) permeability, and inflammatory responses [48–53]. Elevated proinflammatory cytokines tumor necrosis factor-α (TNF-α), interleukin-1 (IL-1), interleukin-6 (IL-6), and interferon-γ (IFN-γ) as well as altered activation of macrophages, T cells, natural killer cells, and other immune cell populations are associated with major comorbidities (including diabetes) (Figs. 1 and 2) for stroke [54].

Thus, it is important to understand how neuroinflammatory mediators following hypoglycemia and diabetes-associated cerebral ischemia produce irreversible CNS injury. This will provide a basis for the development of effective therapies to minimize the extent of damage and improve clinical outcomes.

### Mechanisms of cerebral ischemic damage

The lack of oxygen and glucose during ischemia activates an array of pathways, including bioenergetics failure, loss of cell ion homeostasis, acidosis, increased intracellular calcium levels, glutamate excitotoxicity, reactive oxygen species (ROS)-mediated toxicity, generation of arachidonic acid products, cytokine-mediated cytotoxicity, activation of neuronal nuclear factor kappa-light-chain-enhancer of activated B cells (NFκB) and glial cells, complement activation, disruption of the BBB, and infiltration of leukocytes [55, 56]. Ischemia-induced glutamate excitotoxicity is a pathogenic process that can lead to calcium-mediated neuronal injury and death by generating ROS and nitrogen species, as well as impairing mitochondrial bioenergetic function [57–59]. The resulting oxidative stress causes further damage and may ultimately result in the initiation of pathways that lead to necrotic and apoptotic cell death.

## Mechanisms of damage following cerebral ischemia

### Apoptosis

The process of programmed cell death, *apoptosis*, acts as a defense mechanism to remove damaged, unwanted, or potentially harmful cells. Apoptosis is also termed type I programmed cell death (type I PCD) [60] and is characterized by nuclear condensation and fragmentation, cleavage of chromosomal deoxyribonucleic acid (DNA) into internucleosomal fragments, and the formation of apoptotic bodies. These apoptotic bodies are removed by phagocytosis [61]. Apoptosis after cerebral ischemia can occur *via* intrinsic and extrinsic pathways. The intrinsic pathway is initiated by disruption of mitochondria and secretion of cytochrome *C* which leads to caspase activation which subsequently leads to apoptotic cell death (reviewed in detail in [62]).

The extrinsic pathway involves cell surface receptors and ligands that lead to cell death. Forkhead1, a member of the forkhead family of transcription factors, stimulates the expression of target genes, e.g., Fas ligands (FasL), which are implicated in the extrinsic receptor pathway of caspase 3 activation. FasL binds to Fas death receptors (FasR), which triggers the recruitment of the Fas-associated death domain protein (FADD). FADD binds to procaspase-8 to create a death-inducing signaling cascade (DISC), which activates caspase 8. Activated caspase-8 either mediates cleavage of BH3 interacting-domain death agonist (Bid) to truncated Bid (tBid), which integrates the different death pathways at the mitochondrial checkpoint of apoptosis, or directly activates caspase-3. At the mitochondrial membrane, tBid interacts with Bcl-2 (B cell lymphoma-2)-associated X protein (Bax). Dimerization of tBid and Bax leads to the opening of mitochondrial transition pore, thereby releasing cytochrome *C*, which initiates caspase 3-dependent cell death (reviewed in detail in [62]).

### Necrosis

Necrosis is another major pathway of cell death observed following cerebral ischemia. Morphological characteristics of necrosis include vacuolation of the cytoplasm, breakdown of the plasma membrane, and induction of inflammation around the dying cell by release of cellular contents including lysosomes and proinflammatory molecules [61]. Necrotic cell death in ischemic brain injury occurs via poly(adenosine diphosphate (ADP)-ribose)-polymerase (PARP) and/or calpains. PARP-1 activity following cerebral ischemic injury is high. Absence or inhibition of PARP-1 is shown to lower cerebral ischemic damage in in vivo and in vitro models of cerebral ischemia and/or excitotoxicity [63, 64]. An increased intracellular free Ca^2+^ level activates multiple Ca^2+^-dependent enzymes such as neutral cysteine proteases and calpains [65]. The excessive activation of calpain-induced cytoskeletal protein breakdown leads to structural integrity and disturbances of axonal transport, and finally to necrotic cell death [66]. Reduced cerebral ischemic damage with the calpain inhibitor, Cbz-Val-Phe-H, confirms the role of calpain in cerebral ischemic damage [67].

### Other mechanisms of cell death

Autophagy, a third type of cell death, also contributes to cerebral ischemic damage [68, 69]. Autophagy is also known as type II PCD [70]. An association between autophagy and injury has been demonstrated in experimental model of stroke by Wen et al. [71]. In their study, 3-methyladenine (3-MA; an autophagy inhibitor) treatment significantly lowered infarct volume, brain edema, and motor deficits. These neuroprotective effects were associated with an inhibition of ischemia-induced upregulation of light chain 3-II (LC3-II)—a marker of active autophagosomes and autophagolysosomes. Another study observed that inhibition of autophagy, either by direct inhibitor 3-MA or by indirect inhibitor 2-methoxyestradiol (2ME2) (an inhibitor of hypoxia inducible factor-1α (HIF-1α)) prevented pyramidal neuron death after ischemia [72]. Mice deficient in autophagy-related gene (Atg)7, the gene essential for autophagy induction, showed nearly complete protection against hypoxia-ischemia-induced neuronal death, indicating autophagy as one of the important mechanisms of cell death following hypoxia–ischemia [73]. All these studies demonstrated involvement of autophagy in cerebral ischemic damage.

## Neuroinflammatory mechanism of cell death following cerebral ischemia

### Cellular mediators of inflammation

After cerebral ischemia, neuroinflammation occurs, which is characterized by the accumulation of inflammatory cells and other mediators in the ischemic brain from resident brain cells (activated microglia/macrophages, astrocytes) and infiltrating immune cells (leukocytes). Which subsequently leads to inflammatory injury.

#### Leukocytes/macrophages

The recruitment of leukocytes from the circulation into the extravascular space in the brain is a central feature after ischemia/reperfusion (I/R). The leukocyte population primarily consists of neutrophils, monocytes, and lymphocytes, each of which can contribute to inflammation following ischemia (reviewed in detail in [74]). Monocytes transform into blood-borne macrophages upon activation. Macrophages play a dual role after cerebral ischemia owing to expressions of anti- and proinflammatory mediators. Macrophages exert neurotoxic effects by creating prothrombotic and proinflammatory environment *via* the release of platelet-activating factor, proinflammatory cytokines (TNF-α, IL-1β), and superoxide anions [75]. The macrophages also confer beneficial effects by removing damaged cells *via* phagocytosis [76, 77].

#### Microglia

Microglia are modulators of the immune response in the brain [78, 79]. Once activated, these cells are indistinguishable from circulating macrophages [80]. Activated microglia eliminates foreign organisms by means of phagocytosis. However, microglia when activated following ischemia contributes to ischemic injury *via* production of neuroinflammatory mediators toxic to cells (reviewed in detail in [74, 81]).

#### Astrocytes

Astrocytic activation represents a potentially damaging mechanism following cerebral ischemia by producing inflammatory mediators and cytotoxic molecules such as ROS, nitrogen species, and proteases, among others [82]. Overall, astrocytic activation is involved in damaging consequences following cerebral ischemia.

### Neuroinflammatory response after cerebral ischemia

Cerebral ischemia leads to the activation of microglia and astrocytes as well as mobilization and infiltration of peripheral inflammatory cells into the brain. The development of post-ischemic brain inflammation is coordinated by activation, expression, and secretions of numerous proinflammatory mediators such as cytokines, chemokines, and adhesion molecules from the brain parenchyma and vascular cells, all of which contribute to increased vulnerability of neurons, and causes BBB disruption and further stimulates gliosis, which further leads to cell damage and ultimately death [74, 81]. Lowering ischemic damage by targeting neuroinflammatory pathways is considered one of the important areas of research in recent years.

#### Cytokines

Cytokines are inflammatory mediators produced by leukocytes, macrophages, endothelial cells, and resident cells within the CNS, including glial cells and neurons, in response to a diverse range of injuries. Following cerebral I/R, altered expression of proinflammatory and anti-inflammatory cytokines worsens tissue pathology.

##### Anti-inflammatory cytokines

Interleukin-10 (IL-10): IL-10 inhibits interleukin-1β (IL-1β), TNF-α, and interleukin-8 (IL-8) as well as lowers cytokine receptor expression and receptor activation [83]. Animal studies have confirmed the anticipated neuroprotective role of this anti-inflammatory cytokine in ischemic stroke [84–86]. In in vitro models, IL-10 protects murine cortical and cerebellar neurons from excitotoxic damage and oxygen/glucose deprivation by activating survival pathways [85, 87]. Clinically, lower IL-10 plasma levels have been associated with increased risk of stroke [88]. Collectively, these studies suggest that IL-10 is neuroprotective through indirect effects on proinflammatory pathways.

Transforming growth factor -β (TGF-β): TGF-β1 has been regarded as an important endogenous mediator that responds to ischemic injury in the CNS [89–91]. Studies have shown neuroprotective activity of TGF-β1 against ischemia [92–95]. One recent report demonstrated the anti-inflammatory effect of TGF-β by inhibiting excessive neuroinflammation during the sub-acute phase of brain ischemia [96]. Intra-carotid administration of TGF-β has been shown to reduce the number of circulating neutrophils, which may ameliorate the post-ischemic no-reflow state [97]. TGF-β may also reduce neutrophil adherence to endothelial cells, suppresses the release of potentially harmful oxygen- and nitrogen-derived products, promotes angiogenesis in the penumbral area, and reduces the expression and efficacy of other cytokines such as TNF-α [98]. Thus, knowing the exact mechanisms involved behind neuroprotection played by these anti-inflammatory cytokines may lead to more effective therapies that limit brain injury during ischemia.

##### Proinflammatory cytokines

Interleukin-1 (IL-1): Interleukin-1 is a major mediator of the inflammatory response following ischemia, with potentially neurotoxic effects. There are two isoforms, IL-1α and IL-1β. IL-1 receptor antagonist (IL-1ra) is an endogenous inhibitor of IL-1 [99, 100]. Post-ischemic increase in the levels of IL-1β correlates with larger infarct size. Intraventricular injection of recombinant IL-1β enlarged infarct volume and brain edema as well as increased influx of neutrophils after middle cerebral artery occlusion (MCAO) [101]. The deleterious effects of IL-1 were also demonstrated by Garcia et al [102] and Relton et al [103] who showed that administration of recombinant IL-1 receptor antagonist reduces the severity of neurologic deficits and tissue necrosis in rats subjected to permanent MCAO. The inhibition of IL-1β signaling with IL-1ra has been found to be protective in experimental models of stroke [104]. Recombinant human IL-1 receptor antagonist (rhIL-1ra) was well tolerated and appeared to be safe when administered within 6 h of acute stroke in a clinical trial [105]. IL-1, and in particular, IL-1β plays an important role in brain injury during ischemia. Thus, modulating IL-1β expression may help to reduce the exacerbation of IL-1β-induced ischemic injury.

Tumor necrosis factor-α (TNF-α): TNF-α is a well-known inflammatory factor associated with worsened clinical outcomes after stroke and exacerbations of infarct size in pre-clinical models [106, 107]. TNF-α is increased in the serum of stroke patients between 6 and 12 h after symptom onset [108, 109]. TNF-α levels in cerebrospinal fluid (CSF) and serum of patients with ischemic stroke were markedly increased within 24 h, and this increase in levels of CSF and serum TNF-α was positively correlated with infarct volume [110]. Like IL-1, TNF-α induces adhesion molecule expression in cerebral endothelial cells and promotes neutrophil accumulation and transmigration. In addition, TNF-α stimulates acute-phase protein production, disrupts the BBB, and stimulates the induction of other inflammatory mediators [111].

TNF-α is a pleiotropic cytokine that possesses both neurotoxic and neuroprotective effects [112]. TNF-α is believed to have detrimental roles during the early phase of the inflammatory response while beneficial roles in the later stages [113]. On one hand, blockade of TNF-α reduces infarct volume after permanent MCAO [106]. Similarly, the anti-TNF-α antibody Pl14 and the TNF synthesis inhibitor CNI-1493 also improve behavioral deficits in Lewis rats after stroke [114]. Treatment with the PARP inhibitor PJ34 [115], the proteosome inhibitor MLN519 [116], or the tree-derived compound brazilien [117] is associated with reduced brain TNF-α expression after transient MCAO. All these experimental manipulations reduce the area of infarct and neurological deficits. This indicates a deleterious role of TNF-α in stroke progression in these animal models.

On the other hand, TNF-α pretreatment is neuroprotective against permanent MCAO [118]. Knockout mice deficient in TNF-α receptors have enhanced sensitivity to stroke, with exacerbated neuronal damage [119]. TNF-α can also mediate neuroprotection in other situations. In one study, sodium nitroprusside was used to induce acute nitric oxide excitotoxicity in TNF-α knockout mice. These mice showed dramatic exacerbation of neuronal damage, suggesting that early endogenous TNF-α release after the insult is neuroprotective [120]. In another study, TNF-α-expressing neurons from TNF-α-transgenic mice were strongly protected from apoptosis induced by glutamate, a substance inducing excitotoxicity in primary cortical neurons. Neurons from wild-type mice pretreated with TNF-α were also resistant to excitotoxicity [121]. Further, excitotoxic neuronal death induced by *N*-methyl-d-aspartate (NMDA) is reduced by TNF-α treatment in cultured cortical neurons [122]. Thus, the neurotoxic and neuroprotective effect of TNF-α depends on several factors such as cellular source, activation of TNF-α receptors, timing and threshold of TNF-α released, and factors that stimulate TNF-α signaling.

Interleukin-6 (IL-6): IL-6 is a pleiotropic cytokine. It is unclear whether the overall effect of IL-6 is beneficial or detrimental following cerebral ischemia. The IL-6 level remains elevated starting at 4 h to 2 weeks post ischemia with the peak at 24 h post ischemia [113, 123, 124]. IL-6 stimulates T lymphocyte proliferation and infiltration into the brain leading to increased inflammatory response. However, IL-6 does not contribute to ischemic brain injury as IL-6 can upregulate IL-1ra, and lack of IL-6 (deficient mice) does not affect post-ischemic outcome [125, 126]. Thus, it is unclear whether the overall effect of IL-6 is beneficial or detrimental in the context of stroke, although in clinical studies, serum levels of IL-6 were suggested as a good predictor of in-hospital mortality in patients that had suffered an acute ischemic stroke [127]. Also, high plasma IL-6 levels correlate with the severity of stroke [128].

#### Chemokines

The chemokines are the members of the G-protein-coupled receptor superfamily and are classified by position of cysteine residues [129]. Chemokines and chemokine receptors have been found to be upregulated following ischemia and signal leukocytes to traffic on the inflamed cerebral endothelium [130]. Upregulated expression of several chemokines and their receptors including, C-C motif chemokine ligand-2/monocyte chemoattractant protein-1(CCL-2/MCP-1), C-C motif chemokine ligand-3/macrophage inflammatory protein-1 α (CCL-3/MIP-1α), C-C motif chemokine ligand-5/regulated on activation, normal T cell expressed and secreted (CCL-5/RANTES), C-C motif chemokine ligand-7 (CCL-7), C-X-C motif chemokine ligand-10/interferon inducible protein-10; (CXCL-10/IP-10), C-C motif chemokine ligand-20 (CCL-20), and chemokine receptors C-X-C motif chemokine receptor-4 (CXCR-4) and C-C motif chemokine receptor-6 (CCR-6) following ischemia have been reported earlier [131–136].

Post-ischemic increase in production and release of chemokines (e.g., cytokine-induced neutrophil chemoattractant: CINC, MCP-1, Fracktalkine, macrophage inflammatory protein: MIP-1, etc.), which is suggested to be stimulated by cytokines (especially IL-1β, TNF-α, and IL-6), is responsible for regulation and migration of monocytes, neutrophils, and lymphocytes at the site of inflammation [137–144]. In rats, administration of anti-CINC antibody decreases cerebral edema and infarction, which further supports a role for CINC in mediating neutrophils and demonstrates another therapeutic opportunity [145].

Inhibition of chemokines during ischemic injury is associated with improved outcomes [146], while over-expression of chemokines exacerbates injury through increased recruitment of inflammatory cells [130]. Previous studies have reported that chemokine or chemokine receptor inhibition or deficiency can decrease ischemic brain injury. MCP-1 deficiency in genetically altered mice and the blockade of chemokine receptors, using nonpeptide C-C chemokine receptor antagonist TAK-779, modulated inflammatory responses in the CNS resulting in reduced infarct volume and macrophage accumulation in a stroke model [147, 148], respectively. It has been shown that anti-MCP-1-neutralizing antibody attenuated NMDA-induced brain injury in the striatum and hippocampus [149]. Intracerebroventricular administration of anti-MIP-3α neutralizing antibody reduces transient MCAO-induced infarct size [134]. A pharmacological inhibitor of C-X-C motif chemokine ligand-8 (CXCL-8), repertaxin, is neuroprotective in a rodent model of transient brain ischemia and its beneficial effects have been attributed to the inhibition of neutrophil recruitment and decreased secondary injury [146]. Inhibition of C-X-C motif chemokine receptor-1 (CXCR-1)/-2 receptors by reparixin (acting as a noncompetitive allosteric antagonist of the CXCR-1 and CXCR-2 receptors) protected the brain after MCAO [150]. After 24 h of reperfusion, pretreatment with reparixin significantly reduced myeloperoxidase (MPO) activity and reduced the levels of IL-1β [150]. The administration of SB225002, a CXCR-2 antagonist, was also associated with reduced neutrophil infiltration in the brains of rats 24 h after cerebral I/R, but did not improve outcome. Mice treated with either SB225002 or vehicle had similar motor impairment and infarct volume at 72 h [151]. C-X3-C motif chemokine receptor-1(CX3CR-1) deficiency correlates with improved neurological function following MCAO and suggests that blockade of CX3CR-1/C-X3-C motif chemokine ligand-1 signaling may provide neuroprotection against ischemic injury. In regard to acute CNS injury models (transient and permanent brain ischemia, spinal cord injury), the collective data suggest that the absence of CX3CR-1 significantly reduces ischemic damage and inflammation [152–154]. The ability of chemokines to control precisely the movement of inflammatory cells suggests that chemokines and their receptors might provide novel targets for CNS therapeutic intervention.

#### Matrix metalloproteinases (MMPs)

The MMPs are zinc- and calcium-dependent endopeptidases, identified as matrix-degrading enzymes. MMP-9- and MMP-2-mediated disruption of BBB integrity and neuronal cell death has been suggested following cerebral ischemia [155, 156]. Treatment with MMP-9 inhibitor within 24 h of stroke reduced infarct size at day 14, and this benefit was lost when the treatment was delayed until 72 h. Further delayed in the treatment (until day 7 post-stroke) exacerbated brain pathology [157]. Additionally, broad-spectrum MMP inhibitors such as BB-94 and BB-1101 have been shown to reduce infarct size and restore BBB integrity in rodent stroke models [158, 159]. Although prolonged inhibition of MMP-9 was found to be detrimental to the late recovery phase of stroke [160, 161]. MMP-2 and MMP-9 selective inhibitor SB-3CT reduced infarct size when administered at 6 h of ischemia onset [162]. In human ischemic stroke, active MMP-2 is increased first on days 2–5 compared to active MMP-9, which is elevated up to months after the ischemic episode [163]. The increased plasma MMP-9 level and the presence of MMP-9 in human brain sections after both ischemic and hemorrhagic stroke further support a role for MMP-9 in the pathophysiology of stroke [163, 164]. The available literature suggests that future therapeutics targeting specific MMP inhibition might be beneficial in ischemic stroke.

#### Cell adhesion molecules

Cell adhesion molecules (CAM) are cell-surface proteins that mediate cell–cell and cell–extracellular matrix interactions [165]. Adhesion molecules play a crucial role in the pathophysiology of acute ischemic stroke [166]. The three main groups of CAMs: the selectins, the immunoglobulin gene superfamily, and the integrins play main role in leukocytes and the vascular endothelium interaction [167].

Selectins: Selectins are membrane-bound glycoproteins that are necessary for the initial capture and rolling of leukocytes on the vessel wall during inflammation [168]. There are three selectins, i.e., L- (leukocyte), E- (endothelial), and P- (platelet) selectins and all of them share a common sequence and structural features [169]. Selectins once activated binds with carbohydrate residues [sialyl-LewisX (sLeX)] and participates in tethering and rolling of circulating leukocytes on endothelium. Dysregulated selectin expression contributes to the inflammation [168].

Leukocyte adhesion has been demonstrated in different experimental models of cerebral ischemia and hypoxia [170, 171]. Although L-selectins mediate the initial rolling of leukocytes, their exact involvement in the development of ischemic injury is not known. Blockade of L-selectin with a humanized anti-L-selectin antibody did not lessen the extent of leukocyte adhesion and transmigration into the areas of damage in a rabbit model of transient focal cerebral ischemia [172]. In another study using cerebral I/R model, anti-L-selectin antibodies were found to be effective only when used in combination with tissue plasminogen activators (tPA), which addresses the potential involvement of L-selectin in tissue injury following thrombolytic reperfusion of the ischemic brain [173].

Following cerebral ischemia, P- and E-selectins are highly expressed in the brain. P-selectin can be detected as early as 15 min after reperfusion while E-selectin expression is observed beginning at 2 h after ischemia. The expression of selectins contributes to the early recruitment of circulating cells to the infarct region [174], and blocking their function has neuroprotective effects in certain stroke models [175, 176]. Anti-selectin antibodies or a synthetic analog of sLeX lowers damage following cerebral ischemia [177, 178]. In a model of cerebral I/R, P-selectin knockout mice exhibited a reduction in infarct volume, better functional outcome, and a better return of cerebral blood flow after ischemia [179]. In a permanent ischemia model, P-selectin immunoblockade attenuated both infarct size and brain edema, which were associated with a reduction of leukocyte infiltration [180]. In these studies, the anti-P-selectin antibodies were administered 30 min before the ischemic insult, which lessens the therapeutic value of the observed protection. Overall, it is suggested that antagonizing selectin using either anti-selectin antibodies or anti-selectin peptides is effective in reducing stroke volume.

Immunoglobulin (Ig) superfamily: The immunoglobulin superfamily class of cell adhesion molecules mediates the adhesion of leukocytes to endothelial cells. In terms of leukocyte–endothelial interactions, the Ig superfamily consists of five molecules: intercellular adhesion molecule (ICAM)-1 and ICAM-2, vascular cell adhesion molecule (VCAM)-1, platelet–endothelial cell adhesion molecule-1 (PECAM-1), and mucosal addressin cell adhesion molecule-1 (MAdCAM-1) [181]. After cerebral ischemia, ICAM-1, ICAM-2, VCAM-1, and PECAM-1 have been shown to contribute to the inflammatory response [182, 183]. ICAM-1 expression is an essential step in mediating the firm adhesion of leukocytes in cerebral microvessels after ischemic stroke, and there are several studies that address the contribution of ICAM-1 to cerebral injury after stroke [184–188]. Immunoneutralization or genetic deletion of cell adhesion molecules that mediate leukocyte recruitment reduces tissue injury and brain dysfunction in animal models of focal and global cerebral ischemia (reviewed in [166]).

Studies have shown that ICAM-1-deficient mice have smaller infarcts compared to wild-type mice following focal cerebral ischemia [184, 185]. Similarly, ICAM-1 immunoblockade reduces ischemic brain injury and neutrophil accumulation in both rat and rabbit models of cerebral ischemia [186–188]. These findings help emphasize the critical role of leukocyte adhesion in furthering inflammatory injury following cerebral ischemia. A significant reduction in ischemic lesion was observed in anti-ICAM-1 antibody-treated or ICAM-1 antisense oligonucleotide-treated group following transient MCAO [189, 190].

VCAM-1 is upregulated following stimulation by cytokines (i.e., IL-1 and TNF-α) [191]. However, the role of VCAM-1 in inflammatory injury is not completely understood. Inhibition of VCAM-1 expression was neuroprotective in a model of transient global cerebral ischemia [192], while inhibition of VCAM-1 was not neuroprotective in a focal cerebral ischemia model [193]. Increased plasma and CSF concentrations of soluble ICAM-1 (sICAM-1) and soluble VCAM-1 (sVCAM-1) were measureable in patients shortly following cerebral ischemic events and these concentrations correlated with the severity of injury [194, 195]. Thus, we conclude that future studies involving anti-adhesion therapies in ischemic stroke will provide promising strategies in modulating adhesion properties of post-ischemic cerebral microvasculature and thereby limit brain injury.

Integrins: The integrins respond to a variety of inflammatory mediators, including cytokines, chemokines, and chemoattractants [196]. Integrins are transmembrane surface proteins that consist of a common β-subunit dimerized with a variable α-subunit (cluster of differentiation (CD)11a, CD11b, or CD11c) [197]. The CD11a/CD18 integrin is referred as lymphocyte function-associated antigen-1 (LFA-1), whereas CD11b/CD18 is called leukocyte adhesion receptor macrophage-1 antigen (Mac-1). Upregulated LFA-1 and Mac-1 expression contribute to the severity of ischemic stroke. Mice deficient in Mac-1 showed reduced infarct volume and reduced neutrophil extravasation after cerebral ischemia [198–200]. Blocking CD11b [200, 201] as well as CD18 [202] or both [203, 204] reduces injury from experimental stroke and is associated with decreased neutrophil infiltration. Similarly, mice lacking CD18 exhibited reduced leukocyte adhesion to endothelial cell monolayers and improved cerebral blood flow with less neurological injury and neutrophil accumulation when subjected to experimental stroke [205]. Blocking integrins essential for lymphocyte and monocyte trafficking may also limit damage due to reperfusion injury.

Clinical studies examined the potential of anti-integrin therapies in acute stroke patients. In a phase III trial, stroke patients were treated with humanized anti-Mac-1 antibody (LeukArrest), the first dose within 12 h while the second dose at 60 h post-symptom onset [206]. Another trial was a phase IIb dose escalation study of a non-antibody peptide, recombinant neutrophil inhibiting factor (rNIF) in stroke patients (Acute Stroke Therapy by Inhibition of Neutrophils or ASTIN) administered within 6 h of symptom onset [207]. Both studies were terminated prematurely owing to a lack of effect on predetermined endpoints. In a rabbit model of transient focal ischemia, administration of LeukArrest 20 min post ischemia decreased neutrophil infiltration and reduced neuronal injury (52% reduction) [76, 204]. No beneficial effect was observed in models of permanent stroke [76, 208]. Anti-adhesion molecule strategies using integrins as targets in ischemic stroke have proven more effective following transient, but not permanent ischemia [189, 205, 209].

#### Toll-like receptors (TLRs)

TLRs are a family of pattern recognition receptors that were initially identified for their role in the activation of innate immunity in response to the presence of exogenous microorganisms; however, TLRs also play a role in ischemic injury in the absence of infection [210]. In this setting, TLRs recognize endogenous molecules released during injury. Such endogenous molecules are known as damage-associated molecular patterns (DAMPs). The binding of DAMPs to their respective receptors results in the activation of an inflammatory response that can exacerbate ischemic damage [210]. Upregulated TLRs levels associated with enhanced cell damage and their inhibition/blockade correlated with reduced infarct size following ischemia [211–214]. The involvement of TLRs and their ligands in inflammation-induced neuronal injury following cerebral ischemia is widely reported [111, 211–216]. TLR-4-deficient mice showed reduced infarct size, better outcomes in neurological and behavioral tests, and decreased level of inflammatory mediators following experimental stroke [217–219]. TLR-2 and the TLR-4 mutant mice showed significantly smaller post-stroke brain damage and lower neurological impairments compared with wild-type mice [220]. Thus, modulating TLR-2 and TLR-4 levels protects the brain against ischemia-induced neuronal damage. Clinical studies have also examined the role of TLRs in stroke patients, including those that focus on the association of TLR-4 polymorphisms with the prevalence of stroke [221, 222]. Thus, TLRs appear to be involved in ischemic injury both in experimental models and in clinical studies. These could be potential targets for future studies focusing on therapeutic approach.

## Diabetes and hypoglycemia

Severe hypoglycemia is considered a medical emergency as it causes organ and brain damage. The types of symptoms that depend on duration and severity of hypoglycemia includes autonomic symptoms (sweating, irritability, and tremulousness), cognitive impairment, seizures, and coma. Brain damage, trauma, cardiovascular complications, and death are major complications of severe hypoglycemia [223]. The incidence of hypoglycemia depends on the degree of glycemic control. Threefold increase in incidences of severe hypoglycemia and coma in intensively treated group was observed when compared to conventionally treated group in the Action to Control Cardiovascular Risk in Diabetes (ACCORD) study [224].

The risk of hypoglycemia in randomized controlled trials of glucose regulation in stroke settings has been reported ranging from 7 to 76% [225–230]. The ischemic brain is particularly susceptible to hypoglycemia [231]. In the presence of stroke, it is possible that incidents of hypoglycemia may be mistaken for progressing severity of stroke, given that symptoms of hypoglycemia include impaired cognitive functioning, hemiparesis, seizures, and coma.

Hypoglycemia is proposed to be linked with angina, myocardial infarction, and acute CVD [232–234]. Hypoglycemia causes a cascade of physiologic effects and may induce oxidative stress [235], induce cardiac arrhythmias [236], contribute to sudden cardiac death [236], and cause cerebral ischemic damage [237], presenting several potential mechanisms through which acute and chronic episodes of hypoglycemia may increase CVD risk.

Increased levels of C-reactive protein (CRP), IL-6, IL-8, TNF-α, and endothelin-1 have been shown during hypoglycemia [238, 239]. Wright et al. [240] and Gogitidze Joy et al. [32] confirmed that hypoglycemia induced an increase in proinflammatory mediators and platelet activation, and has an inhibitory effect on fibrinolytic mechanisms. Hypoglycemia also increases production of vascular endothelial growth factor (VEGF), increases platelet and neutrophil activation leading to endothelial dysfunction, and decreased vasodilation, resulting in increased risk for CVD events [241]. Furthermore, IL-1 has been shown to increase the severity of hypoglycemia [242]. Moderate hypoglycemia acutely increases circulating levels of plasminogen activator inhibitor-1 (PAI-1), VEGF, vascular adhesion molecules (VCAM, ICAM, E-selectin), IL-6, and markers of platelet activation (P-selectin) in T1D patients and healthy individuals [32]. Thus, hypoglycemia can result in complex vascular effects including activation of prothrombotic, proinflammatory, and proatherogenic mechanisms in T1D patients and healthy individuals. In addition, a link has been made between low glucose levels and the unexpected sudden death in T1D patients without CVD, also known as “dead in bed” syndrome [243].

Recurrent severe hypoglycemia results in brain damage [244], with preferential vulnerability in the cerebral cortex and hippocampus [244–246]. Evidence suggests that neuronal damage resulting from hypoglycemia is enhanced in diabetic compared to non-diabetic brains [245]. Hypoglycemia causes a loss of ionic homeostasis or increase in ROS that can further lead to neuronal inflammation and death [246].

## Impact of hypoglycemia in the diabetic brain

Hypoglycemia is of major concern in diabetes as it leads to severe impairment of CNS function. Severe and/or long duration hypoglycemia may result in severe morbidity and even death. Repeated episodes of hypoglycemia are suggested to increase the risk of atherosclerosis [247]. Acute hypoglycemia results in endothelial dysfunction, vasoconstriction, white blood cell activation, and release of inflammatory mediators including cytokines *via* sympathoadrenal stimulation and release of counter-regulatory hormones [32]. All these changes increase the risk of myocardial and cerebral ischemia [240].

Recurrent/moderate hypoglycemia also aggravates post-ischemic brain damage in diabetic rats [53]. In this study, rats treated with insulin and exposed to recurrent hypoglycemic episodes experienced a 44% increase in neuronal death compared with rats similarly treated with insulin but not exposed to hypoglycemia, demonstrating that prior exposure to recurrent hypoglycemia can lead to more extensive cerebral ischemic damage. Relatively severe recurrent hypoglycemia itself induces neuronal death in the CA1 hippocampus and cortex of streptozotocin-induced diabetic rats [248, 249].

Bree and collaborators [245] showed that insulin-induced severe hypoglycemia in normal animals elicits brain damage in the cortex, cornus ammonis (CA)1, and CA3 hippocampal regions, and that the diabetic condition increases the vulnerability to neuronal death in these specific brain areas. These results suggest that diabetes can be a critical factor aggravating neuronal damage in hypoglycemia.

Decreased cognitive function can also lead to an increased risk of hypoglycemia and CVD events, and thus mortality [250]. In a study examining magnetic resonance imaging of the brain in a cohort of 22 patients with T1D, brain abnormalities were more common in patients with T1D who had a history of repeated (five or more) hypoglycemic episodes [251]. In some of the strongest evidence to date of the detrimental effects of hypoglycemia on cognitive function, Whitmer et al. [252] investigated the association of hospitalization or emergency department visits for hypoglycemia and dementia development in older adults with T2D. They reported a dose/response relationship between the number of hypoglycemia episodes and the risk for developing dementia.

## Inflammatory response in diabetes/hyperglycemia

Increased systemic and cerebrovascular inflammation is one of the key pathophysiological features in diabetes and its vascular complications [253, 254]. Though the etiology of diabetic complications is multifactorial, chronic inflammation is thought to play a critical role [255, 256]. Key mechanisms of hyperglycemia-induced inflammation include NFkB-dependent production of proinflammatory cytokines, TLR expression, increased oxidative stress, and inflammasome activation [256–259].

Increased expression of proinflammatory cytokines has been demonstrated in diabetes (reviewed in [260]). Proinflammatory cytokines IL-12 and IL-18 were shown to be elevated in serum of diabetic patients compared to healthy subjects and were positively associated with CRP, which is one of the most important biomarkers of chronic inflammation [261, 262]. CRP itself exerts direct proinflammatory effects on human endothelial cells, inducing the expression of adhesion molecules [263]. IL-12 and IL-18 have been shown to exert strong proinflammatory activity that synergize with each other, as well as with TNF-α or IL-1 [264]. NFκB controls the induction of many inflammatory genes. During hyperglycemia, NFκB is rapidly and dramatically activated in vascular cells resulting in a subsequent increase in leukocyte adhesion and transcription of proinflammatory cytokines [41]. A significant increase in expression of proinflammatory cytokines (TNF-α, IL-6, and IL-1β), followed by activation of NFkB and signal transducer activator of transcription 3 (STAT3) inflammatory pathways, was reported in cultured astrocytes treated with high glucose [265]. Under diabetic conditions, hyperglycemia also causes inflammatory reactions in other organs and tissues in vivo [266, 267]. It has been reported that high glucose in vitro can cause ROS production and expression of proinflammatory cytokines and chemokines in a variety of cells [268–270]. Expression of adhesion molecules on endothelial cells of both hyperglycemic and diabetic animals, and patients with diabetes, is enhanced compared to normal controls [271].

TLRs play an important role in human and animal model of diabetes. Mice with an inactive TLR-4 gene were significantly less prone to diet-induced insulin resistance [272, 273]. Likewise, inhibition of TLR-2 function in mice exposed to a high-fat diet led to improved sensitivity and decreased activation of proinflammatory pathways [274]. Furthermore, polymorphisms in TLRs and in members of TLR downstream signaling pathways that encode hyper- or hypoactive responses predict the development of T1D and T2D [275, 276]. TLR ligands activate B cell cytokine production, most significantly IL-8, in diabetes mellitus vs. non-diabetic donors [277]. The circulating levels of danger molecules including the high-mobility group box-1 (HMGB-1), heat shock proteins, and hyaluronan that activates TLR signals [278] are known to be increased in T2D patients [258]. Potential roles for TLR-2 and TLR-4 in the pathology of diabetes have been demonstrated recently (reviewed in detail in [279]).

Emerging evidence suggests that activation of the nucleotide-binding and oligomerization domain-like receptor family pyrin domain-containing 3 (NLRP3) inflammasome leads to the maturation and secretion of IL-1β and is involved in the pathogenic mechanisms of obesity-induced inflammation, insulin resistance, and diabetes development [280]. Obesity-induced danger signals have been reported to activate the NLRP3 inflammasome and induce the production of IL-1β in adipose tissue in T2D patients and in mice fed a high-fat diet [281]. Circulating levels of CXCL-10 and CCL-2, as well as IFN-γ mRNA (messenger ribonucleic acid) and protein levels in adipose tissue were significantly reduced in NLRP3-deficient mice, suggesting that the NLRP3 inflammasome plays a role in the macrophage-T cell interactions that are associated with sustained levels of chronic inflammation in obesity-induced metabolic diseases [281]. Moreover, the saturated fatty acid palmitate induces activation of the NLRP3 inflammasome in hematopoietic cells, which is responsible for the impairment of insulin signaling and inhibition of glucose tolerance in mice [282].

## Inflammatory response in hypoglycemia

Recurrent/moderate hypoglycemia induces oxidative injury in hippocampal dendrites, and microglial activation in hippocampus and cerebral cortex [248]. They observed oxidative damage, as assessed by the lipoperoxidation product 4-hidroxynonenal, in the hippocampal CA1 dendritic layer and microglial activation. The degree of microglial activation in the hippocampus of ﻿recurrent/mode﻿rate hypo﻿glycemia﻿-exposed﻿ diabetic rats was 194% higher than in normoglycemic rats ﻿exposed to﻿ recurrent/moderate hypoglycemia [248]. This study confirmed that inflammatory responses are also induced after recurrent/moderate hypoglycemia. Microglial activation is induced in severe hypoglycemia and contributes to neuronal injury by releasing neurotoxic substances, including superoxide, nitric oxide, and metalloproteinases [283–285]. Activation of microglia appears to play a role in the neutrophil infiltration and recruitment which in turn contributes to brain damage [286, 287]. Increased number of infiltrating neutrophils in hypoglycemia vulnerable brain regions following hypoglycemic brain injury suggests its potential role in hypoglycemic brain injury [288].

In another study by Cardoso et al. [289], recurrent hypoglycemia (twice daily for 2 weeks) in streptozotocin-induced diabetic rats potentiated an increase in lipid peroxidation and a decrease in aconitase activity, used as an index of oxidative stress, in mitochondria from diabetic animals. Previous findings showed that recurrent hypoglycemia differentially alters mitochondrial bioenergetics and the antioxidant defense response in the cortex and the hippocampus, the hippocampus being most affected. Limiting ROS production and restoring blood glucose to levels not exceeding the physiological range prevents neuronal death [31]. On the other hand, the administration of pyruvate and lactate in combination with glucose reduces the death of hippocampal neurons [288, 290, 291]. This finding suggests the therapeutic potential of antioxidants, lactate, and pyruvate administration combined with glucose to limit the adverse consequences of glucose reperfusion. On the other hand, it has been recently shown that the administration of minocycline to rats 6 h after hypoglycemic coma and daily for a week results in reduced microglial reactivity, neuronal death, and cognitive impairment [288]. Further investigation is needed to extrapolate these findings to clinical practice.

## Cerebral ischemia-induced inflammatory response in the diabetic brain

Diabetes continues to expand rapidly in the USA. Worldwide, it is projected that diabetes will affect 439 million people by the year 2030 [292]. As mentioned above, diabetes is a predisposing risk factor for cerebrovascular diseases and increases stroke incidence. In humans, diabetes increases the risk of stroke incidence as well as post-stroke mortality [293–295]. Diabetes duration has also been shown to increase the risk of ischemic stroke. With every year of diabetes, the risk is increased by 3% and triples with diabetes of more than 10 years [296]. Diabetes predisposes humans to stroke, and stroke-induced brain damage is known to be exacerbated by poor functional recovery in these patients [297]. Several clinical studies indicated that patients with diabetes had poorer outcomes following stroke [298–302].

Diabetic patients have a higher risk of stroke compared with non-diabetic patients [294, 295]. Although >30% of stroke sufferers are known to be diabetic, the mechanisms that are responsible for the increased post-ischemic brain damage in this population are understudied. Oxidative stress and inflammation play a central role in tissue damage in streptozotocin-induced diabetes [303, 304]. In addition, diabetic patients had significantly increased levels of acute phase proteins and proinflammatory cytokines such as TNF-α and IL-1, compared to non-diabetic controls [305]. More recently, Hwang et al. [306] demonstrated microglial activation and expression of proinflammatory cytokines, such as IFN-γ and IL-1β in the hippocampus of diabetic rats.

The experimental studies have evaluated the effect of diabetes on stroke outcome in T1D and T2D models. The post-ischemic brain damage was exacerbated in T1D rodents following global or focal ischemia [52, 297, 307–310]. The exacerbated edema and infarction, worsened neurological status, and increased mortality have also been observed in T2D models following ischemia [311–314]. A study by Yeung et al. showed that exacerbated post-ischemic pathological symptoms observed in db/db mice are alleviated by knocking out the enzyme of polyol pathway (aldose reductase) that converts glucose to sorbitol and further metabolizes to fructose [315]. Uncontrolled inflammation during the acute period after stroke is a major mediator of cerebrovascular failure and brain damage [316]. Increased expression of cell adhesion molecules enabling the extravasation of white blood cells, and further induction of proinflammatory transcription factors and other inflammatory genes are thought to be major mediators of post-ischemic inflammation [74]. Previously published literature demonstrated the increased expression of ICAM and proinflammatory cytokines in diabetic animals after cerebral ischemia/reperfusion [317–320]. At post-translational levels, IL-1β and cyclooxygenase-2 (COX-2) expressions were significantly higher following hyperglycemic ischemia than hyperglycemic shams [321]. Lin et al. demonstrated that hyperglycemia triggered early, massive deposition of neutrophils in the post-ischemic brain, which exacerbated injury [322]. It has been reported that the expression of ICAM-1 and the infiltration of neutrophils into ischemic tissue are closely correlated with the severity of ischemic brain damage [323]. The gene expression of IL-1β, IL-6, MIP-1α, MCP-1, P-selectin, and E-selectin was much higher in the diabetic mouse brain compared to normoglycemic mouse brain at 12 h of reperfusion following transient MCAO [52]. In another study, diabetic rats had an increased basal level of IL-1β and TNF-α, and inflammatory mediators COX-2 and inducible nitric oxide synthase (iNOS) expressions as compared to that of non-diabetic rats. Transient MCAO increased the gene expression of these cytokines and enzymes, which was remarkably accelerated and augmented by diabetes [324]. Furthermore, this group showed increased expression of MPO and ICAM-1, which are hallmarks of neutrophil, and macrophage/microglia activation and exacerbation in the diabetic rat brain, indicating exacerbation of inflammatory responses in ischemic injury [324]. Enhanced activation of NFκB in the diabetic brain mediated this increased production of proinflammatory cytokines and enzymes [324]. NFκB is a potent inducer of inflammatory processes through its upregulation of the gene expression of proinflammatory cytokines and chemokines such as IL-1β, IL-6, interleukin-17 (IL-17), TNF-α, CRPs, MCP-1, CCL-2, and CXC [325]. The transcription factor NFκB assumes a key role in cerebral ischemia and regulates apoptosis and inflammation [326]. Thus, activation of NFκB is crucial for the inflammatory responses leading to gene expression of proinflammatory cytokines and mediators in immunocytes [326]. Inhibition of NFκB represents a treatment strategy in ischemic stroke [327].

Thus, the exacerbated inflammation might be a contributing factor to the increased post-stroke brain damage observed in the diabetic brain (Figs. 1 and 2). Furthermore, the macrophages and neutrophils release oxygen and nitrogen free radicals which are extremely toxic to neurons. Studies indicate that the extent of stroke-induced brain injury is influenced by the systemic inflammation. It has been shown that increased peripheral inflammation, at the time of stroke, aggravates ischemic injury [328]. Diabetic mice are known to manifest systemic inflammation as well as impaired ability to curtail inflammation [329]. Several proinflammatory proteins including MCP-1 and IL-6 are elevated in the plasma of diabetic patients [330, 331]. The critical role of MCP-1 in the diabetic condition has been demonstrated in studies showing that its overexpression in adipocytes leads to tissue inflammation and insulin resistance, while mice deficient in MCP-1 or its receptor C-C motif chemokine receptor-2 (CCR-2) reverse the condition [332–334]. More recently, Kim et al. [335] demonstrated that in the diabetic condition, acute inflammatory responses are perturbed in the brain following stroke and in the macrophages after lipopolysaccharide stimulation, and these alterations are associated with the exacerbation of stroke-induced injury [335]. Interestingly, diabetic mice were found to display reduced inflammatory cytokine expression and microglial activation, and delayed wound healing [312]. Microglial activation and the release of chemokines and cytokines are critical steps in eliciting inflammatory responses. The inability to mount a proper host immune response immediately after cerebral ischemia in diabetic microglia causes an extended inflammatory phase, which leads to a prolonged infiltration of peripheral immune cells and worsened ischemic injury [335]. The early blunted inflammatory response of MCP-1, IL-6, and CCR-2 in the diabetic mouse brain was reported at 6 h post ischemia [335]. Collectively, the data from this study suggest that early inflammatory responses in the diabetic brain are deregulated, and the alteration is associated with the exacerbation of stroke-induced injury.

An attenuated stroke-induced inflammatory response has been demonstrated in diabetic conditions [312, 313]. Treatment of obese diabetic mice with the peroxisome proliferator-activated receptor γ (PPARγ) agonist darglitazone, for 7 days before induction of hypoxia–ischemia, reduced infarct size and suppressed inflammatory response at 8 and 24 h after ischemia onset [312, 313]. Animal studies have shown that MMP plays an important role in cerebrovascular damage following permanent focal stroke in diabetic rats [336, 337]. A greater MMP-9 activity was found in diabetic rats following stroke [307, 336].

HMGB-1 is a novel player in the ischemic brain [215]. Diabetes significantly increased serum HMGB level and induced worse functional outcome after stroke compared to non-diabetic rats [338]. Diabetes exacerbates systemic inflammation as evidenced by higher serum HMGB-1 in the rat systemic inflammation model [339]. HMGB-1 signaling promotes chemotaxis and production of cytokines in a process that involves the activation of NFκB [340]. Moreover, it has been reported that extracellular HMGB-1 is involved in BBB disruption during the early phase of ischemic stroke [341]. Downregulation of HMGB-1 and NFκB expression protected rat brains against focal ischemia. Suppression of the release of HMGB-1 in astrocytes leads to the attenuation of neuroinflammation, preventing the necrosis of ischemic astrocytes and NFκB expression [342]. Inhibition of the upregulation of HMGB-1 and NFκB at the early stage brings great benefits to cerebral ischemia.

Dysregulated expression of stromal cell-derived factor (SDF)-1α and CXCR-4 has been reported in the diabetic mice brain at baseline and following ischemic stroke [343]. The SDF-1α/CXCR-4 axis is believed to play an important role in recruiting progenitor cells into ischemic tissue. It triggers many intracellular proliferation and anti-apoptosis signals, such as mitogen-activated protein kinase (MAPK), phosphatidylinositol 3-kinase (PI3K), and the serine/threonine Kinase Akt [344]. Therefore, SDF-1α/CXCR-4 is a potential target for promoting repair in wound and ischemic injury.

Overall, diabetes and hypoglycemia aggravates brain damage after ischemic stroke through enhancement of the neuroinflammatory signaling cascade, particularly by the activation of microglia/macrophages, leukocytes, adhesion molecules, upregulation/accumulation of some specific proinflammatory cytokines, MMPs, TLRs, and other immune mediators at the site of injury. All these immune mediators directly or indirectly contribute to further activation of cell death pathways (Figs. 1 and 2).

## Conclusions

Diabetes is a crucial risk factor for stroke. Stroke outcomes are significantly worse among diabetic patients, resulting in increased mortality as well as neurological and functional disabilities. Stroke risk in patients with diabetes is two- to sixfold higher than age-matched controls. Increased incidence of hypoglycemia is the inevitable effect of treatment for aggressively tight glycemic control in diabetes, and is prevalent among both T1D and T2D patients. Studies have shown that diabetes and its associated hypoglycemia exacerbate cerebral ischemic damage in experimental animals. Understanding the mechanisms involved in aggravating neuroinflammatory injury following cerebral ischemia in diabetes and associated hypoglycemia is important. Suppressing potential candidates involved in enhancing neuroinflammatory response may help reduce stroke severity and promote recovery in diabetic/hypoglycemic conditions. An increasing number of studies demonstrate the role of inflammatory mediators in modulating stroke outcome in animal models of T1D and T2D. Thus, targeting inflammatory mediators for future therapeutic strategy in diabetes and its associated hypoglycemic complications appears important. Better understanding of inflammatory pathways involved in diabetes, diabetes-associated hypoglycemia, and diabetic cerebral ischemia may provide unique pharmacological targets for the treatment and/or prevention of hypoglycemia and diabetes-associated stroke damage.

## Abbreviations

2ME2: 2-Methoxyestradiol
3-MA: 3-Methyladenine
ACCORD: The Action to Control Cardiovascular Risk in Diabetes
ADP: Adenosine diphosphate
ASTIN: Acute Stroke Therapy by Inhibition of Neutrophils
Atg: Autophagy-related gene
Bax: Bcl-2-associated X protein
BBB: Blood–brain barrier
Bcl-2: B cell lymphoma-2
Bid: BH3 interacting-domain death agonist
CA: Cornus ammonis
CAM: Cell adhesion molecules
CCL-2: C-C motif chemokine ligand-2
CCL-20: C-C motif chemokine ligand-20
CCL-3: C-C motif chemokine ligand-3
CCL-5: C-C motif chemokine ligand-5
CCL-7: C-C motif chemokine ligand-7
CCR-2: C-C motif chemokine receptor-2
CCR-6: C-C motif chemokine receptor-6
CD: Cluster of differentiation
CGM: Continuous glucose monitoring
CINC: Cytokine-induced neutrophil chemoattractant
CNS: Central nervous system
COX-2: Cyclooxygenase-2
CRP: C-reactive protein
CSF: Cerebrospinal fluid
CVD: Cardiovascular disease
CX3CR-1: C-X3-C motif chemokine receptor-1
CXCL-10: C-X-C motif chemokine ligand-10
CXCL-8: C-X-C motif chemokine ligand-8
CXCR-1: C-X-C motif chemokine receptor-1
CXCR-2: C-X-C motif chemokine receptor-2
CXCR-4: C-X-C motif chemokine receptor-4
DAMPs: Damage-associated molecular patterns
DISC: Death-inducing signaling cascade
DNA: Deoxyribonucleic acid
E: Endothelial
FADD: Fas-associated death domain protein
FasL: Fas ligands
FasR: Fas death receptors
HIF-1α: Hypoxia inducible factor-1α
HMGB-1: High mobility group box-1
I/R: Ischemia/reperfusion
ICAM: Intracellular adhesion molecule
ICAM-1: Intracellular adhesion molecule-1
IFN-γ: Interferon-γ
Ig: Immunoglobulin
IL-1: Interleukin-1
IL-10: Interleukin-10
IL-17: Interleukin-17
IL-1ra: IL-1 receptor antagonist
IL-1β: Interleukin-1β
IL-6: Interleukin-6
IL-8: Interleukin-8
iNOS: Inducible nitric oxide synthase
IP-10: Interferon-inducible protein-10
L: Leukocyte
LC3-II: Light chain 3-II
LFA-1: Lymphocyte function-associated antigen-1
Mac-1: Macrophage-1 antigen
MAdCAM-1: Mucosal addressin cell adhesion molecule-1
MAPK: Mitogen-activated protein kinase
MCAO: Middle cerebral artery occlusion
MCP-1: Monocyte chemoattractant protein-1
MHC: Major histocompatibility complex
MIP-1: Macrophage inflammatory protein-1
MIP-1α: Macrophage inflammatory protein-1 α
MMPs: Matrix metalloproteinases
MPO: Myeloperoxidase
mRNA: Messenger ribonucleic acid
NFkB: Nuclear factor kappa-light-chain-enhancer B cells
NLRP3: Nucleotide-binding and oligomerization domain-like receptor family pyrin domain-containing 3
NMDA: *N*-methyl-d-aspartate
P: Platelet
PAI-1: Plasminogen activator inhibitor-1
PARP: Poly(ADP)-ribose-polymerase
PECAM-1: Platelet-endothelial cell adhesion molecule-1
PI3K: Phosphatidylinositol 3-kinase
PPARγ: Peroxisome proliferator-activated receptor γ
RANTES: Regulated on activation, normal T cell expressed and secreted
rhIL-1Ra: Recombinant human interleukin-1 receptor antagonist
rNIF: Recombinant neutrophil inhibiting factor
ROS: Reactive oxygen species
SDF: Stromal cell-derived factor
sICAM-1: Soluble ICAM-1
sLeX: Sialyl-LewisX
STAT3: Signal transducer activator of transcription 3
sVCAM-1: Soluble VCAM-1
T1D: Type 1 diabetes
T2D: Type 2 diabetes
tBid: Truncated Bid
TGF-β: Transforming growth factor-β
TLRs: Toll-like receptors
TNF-α: Tumor necrosis factor-α
tPA: Tissue plasminogen activators
type I PCD: Type I programmed cell death
VCAM: Vascular cell adhesion molecule
VEGF: Vascular endothelial growth factor
